# Sensitivity Analysis in the Problem of the Impact of an External Heat Impulse on Oxygen Distribution in Biological Tissue

**DOI:** 10.3390/ma18112425

**Published:** 2025-05-22

**Authors:** Marek Jasiński, Maria Zadoń

**Affiliations:** Department of Computational Mechanics and Engineering, Silesian University of Technology, Konarskiego 18A, 44-100 Gliwice, Poland; marek.jasinski@polsl.pl

**Keywords:** bioheat transfer model, oxygen distribution model, oxygen dissociation curve, sensitivity analysis, shooting method, finite difference method

## Abstract

During the exposure of biological tissue to an external heat impulse (both controlled as in various types of thermotherapy and uncontrolled related to thermal burns), processes occur related to changes in its parameters, especially perfusion, and thus the transport of oxygen to the tissue. This paper presents a combined model of bioheat transfer and oxygen distribution in tissue. The latter was based on Krogh’s cylinder concept, taking into account the Hill oxygen dissociation curve. The variable value of the perfusion coefficient is shown to affect the value of blood velocity in the capillary and, therefore, the distribution of partial pressure in the tissue. A sensitivity analysis was performed for the oxygen distribution model using the direct method for seven parameters present in its mathematical description. The results show that a 10% change in the values of all parameters leads to changes in the partial oxygen pressure exceeding 8 mmHg, and for the reduced value of the oxygen inlet pressure, the largest changes in the partial oxygen pressure within the Krogh cylinder model occur near the outlet capillary. In the stage of numerical realization, the finite difference method and the shooting method were used.

## 1. Introduction

Phenomena in living organisms are generally complex because of the couplings between different scales at which interactions between tissue structures and biochemical reactions take place. An additional difficulty is always individual diversity, which often influences ambiguous estimates or wide ranges of tissue parameter values. An example of a complex response of a biological tissue can be its response to an external heat stimulus. With a sufficiently high temperature and exposure time, thermal burns occur, which in extreme situations can mean irreversible tissue damage and even charring. However, between native tissue and thermally damaged tissue, there is a wide spectrum of phenomena associated with raising the temperature of the tissue [1,2,3,4].

It is known that under the influence of temperature, the value of tissue perfusion changes. Since one of the functions of the circulatory system is to supply oxygen to tissues, it can be concluded that an external heat source also interferes with this process [4,5,6,7].

The transport of oxygen from the blood to the tissues takes place in the capillaries, the smallest blood vessels, and the oxygen content in the blood has two forms: chemical bonds that form oxyhemoglobin and free molecules. The parameter that describes the concentration of the latter is the oxygen partial pressure, whereas the relationship between oxygen hemoglobin saturation and oxygen partial pressure is described by the oxyhemoglobin dissociation curve (ODC) [8,9,10,11,12].

Mathematical models of oxygen distribution are usually derived from the concept of the Krogh cylinder, which was adopted and developed over time in many works on muscle work during exercise, the presence of tumor tissue in the body, the process of angiogenesis, and various therapies [7,8,10,11,12]. A model takes into account the capillary and the surrounding cylindrical area of the tissue, with the radius of the cylinder assumed to be such as to ensure the delivery of oxygen to the entire cylinder [6,8,10,11,13].

There are several bioheat transfer equations: the Pennes equation, the Cattaneo–Vernotte equation, and the dual-phase lag model, in which the presence of blood vessels and metabolic phenomena is taken into account through appropriate source heat functions [2,3,14,15,16,17,18,19,20,21,22,23]. Additionally, various functions are additionally adopted to account for changes in thermophysical parameters depending on temperature and/or the degree of thermal damage. However, to estimate the degree of thermal damage, the most commonly used is the so-called Arrhenius scheme, which assumes an irreversible increase in thermal damage as a function of temperature [4,5,13,24].

There are different approaches to studying the aforementioned differences in tissue parameter values. Among other things, interval or fuzzy arithmetic [25,26] has been used for this purpose, and one way is also using sensitivity analysis [27,28,29]. There is fairly extensive literature related to the application of sensitivity analysis methods to bioheat transfer tasks [30,31,32,33,34]. There is much less use of sensitivity analysis methods in areas related to modeling oxygen distribution, so it can be said that the current work fills a gap in this area [35,36].

**Figure 1:**
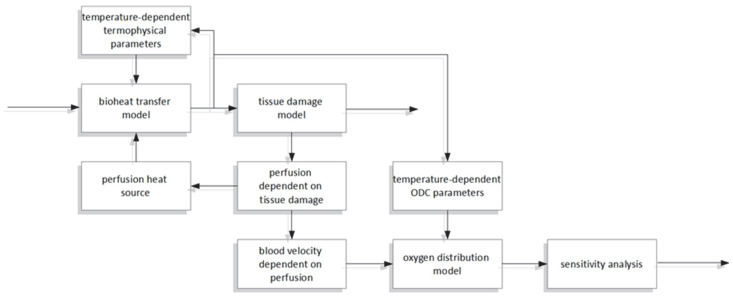
The scheme of the relationships between the particular parts of the analysis.

This work concerns a combined bioheat and oxygen distribution model, with sensitivity analysis performed for the parameters of the latter. Bioheat analysis is based on the Pennes bioheat transfer equation. An Arrhenius scheme was adopted like a thermal damage model, and functions of tissue parameters allowing them to be treated as temperature-dependent (the thermal conductivity and the volumetric specific heat) or thermal damage-dependent (the perfusion coefficient) were also assumed. The thermal model is combined with an axisymmetric model of oxygen distribution in the form of a Krogh cylinder. The oxygen distribution model is linked to the thermal model through the relationship between the blood velocity in the capillary and the perfusion coefficient. The Bohr effect, that is, the shift in the ODC resulting from a change in temperature, was also taken into account. A direct method of sensitivity analysis was used to determine the parameters of the sensitivity function of the oxygen distribution model. The scheme of the relationships between the particular parts of the analysis is presented in Figure 1. The correctness of the mathematical models was verified on available analytical solutions and data from the literature.

## 2. Materials and Methods

### 2.1. Governing Equations

**Figure 2:**
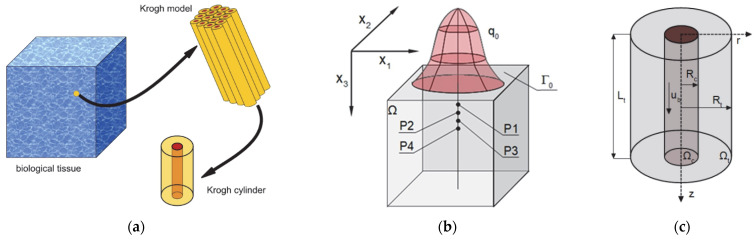
The idea of the Krogh model (**a**) and the domains considered for the bioheat transfer (**b**) and the oxygen distribution model (**c**).

The complete model considered in this work includes a bioheat transfer model and a coupled oxygen distribution model (Figure 2). The first is the 3D domain of biological soft tissue, which is affected at the upper boundary Γ_0_ by an external irregular heat flux *q*_0_. On the other hand, there is a Krogh cylinder, corresponding to the cylindrical area of tissue around a capillary. It should be noted that due to the much smaller size of the Krogh cylinder compared to the thermal model (centimeters vs. micrometers), this type of model does not consider the complete vascular structure but only uses an individual cylinder to represent the average conditions in a certain area of the tissue [6,10]. This is in line with the assumptions of the Krogh model, according to which the tissue is composed of adjacent cylinders, with a diameter large enough so that oxygen transport between the cylinders does not occur, while it is also important that the supply of oxygen to a single cylinder is possible through a capillary running through it (Figure 2) [6,8,12].

The connection between the thermal model and the oxygen distribution model is based on the presence of blood in the tissue. In the thermal model, it is described by a perfusion coefficient, which, from a practical point of view, is often determined by knowing the number of capillaries in a cross section or a certain volume of tissue (depending on the adopted method). Thus, there is a relationship between the perfusion coefficient and the dimensions of the capillary and the velocity of blood in the capillary.

The transient bioheat problem and steady-state problem for the oxygen distribution model were considered. The highlighted points P1–P4 in Figure 2b are the points for which the results of the bioheat analysis, including the perfusion coefficient history, will be presented. Next, for selected time steps, selected point calculations will be carried out using the oxygen distribution model.

Bioheat transfer in the tissue domain Ω is described by the Pennes equation supplemented with the appropriate boundary and initial conditions [7,16,17]:

(1) $$\begin{array}{cc} x\in \Omega : & c\dot{T}=\nabla \left(\lambda \nabla T\right)+Q_{met}+c_{B}w\left(T_{B}-T\right)\\ x\in \Gamma_{0}: & \left\{\begin{array}{cc} q=q_{0}=q_{0,max}\exp\left(-\frac{2(x_{1}^{2}+x_{2}^{2})}{r_{imp}^{2}}\right), & \mathrm{for}\;t\leq t_{exp},\\ q=0, & \mathrm{for}\;t>t_{exp} \end{array}\right.\\ x\in \Gamma_{c}: & q=0\\ t=0: & T=T_{init} \end{array}$$

where λ [W m^−1^ K^−1^] is the thermal conductivity; *c* and *c_B_* [J m^−3^ K^−1^] are the volumetric specific heat of tissue and blood, respectively; *Q_met_* [W m^−3^] is the metabolic heat source; *T_B_* corresponds to the arterial blood temperature; *w* [s^−1^] is the perfusion coefficient; *q*_0_ and *q*_0,max_ [W m^−2^] are the boundary heat flux and the maximal value of the heat flux, respectively; *r_imp_* is the radius of the impulse; *t_exp_* [s] is the exposure time, while *T_init_* denotes the initial distribution of temperature; and x_1_, x_2_, and x_3_ are the cartesian system coordinates (cf. Figure 2b). It should be noted that the adopted external heat impulse in the form of a heat flux can arise from a variety of causes (hot liquid, heater, flame) and can involve both controlled (as in various thermotherapies) and uncontrolled thermal effects on the tissue.

For the axisymmetric Krogh cylinder model, separate equations were adopted for the radial and axial directions (cf. Figure 2). For the radial direction [6,7,13],

(2) $$\begin{array}{ccc} r\in \Omega_{t}: & K_{t}\frac{1}{r}\frac{d}{dr}\left(r\frac{dP_{t}}{dr}\right)=M_{t}(P_{t}), & M_{t}(P_{t})=\frac{M_{0}P_{t}}{P_{crit}+P_{t}}\\ r=R_{c}: & 2\pi R_{c}K_{t}\frac{dP_{t}}{dr}=-k\left(P_{b}-P_{t}\right) & \\ r=R_{t}: & \frac{dP_{t}}{dr}=0 & \end{array}$$

where *P_t_* and *P_b_* [mmHg] are the partial oxygen pressure in tissue and blood, respectively; *K_t_* [(cm^2^ s^−1^) (mol cm^−3^ mmHg^−1^)] is the Krogh diffusion coefficient; *M*_0_ [mol cm^−3^ s^−1^] is the oxygen demand; *P_crit_* [mmHg] is the partial pressure that corresponds to half maximum oxygen consumption; *k* [(cm^2^ s^−1^) (mol cm^−3^ mmHg^−1^)] is the mass transfer coefficient. Note that component *M_t_* (*P_t_*) is oxygen consumption in the tissue, which is assumed to be the Michaelis–Menten kinetics.

For the axial direction in the capillary region, an equation was assumed describing the change in the partial pressure of oxygen *P_b_* [37]:

(3) $$\begin{array}{cc} z\in \Omega_{c}: & \pi R_{c}^{2}u_{b}\kappa_{b}\frac{d\left[S_{Hb}(P_{b})\right]}{dz}=-k\left(P_{b}-P_{t}\right)\\ z=0: & P_{b}=P_{b\;inlet} \end{array}$$

where *u_b_* [cm s^−1^] denotes the blood velocity in capillary, and κ*_b_* [mol cm^−3^_blood_] is the oxygen carrying a capacity of blood at 100% saturation, while *S_Hb_* is the saturation of oxyhemoglobin, determined by the Hill oxyhemoglobin dissociation curve (ODC). Once the *S_Hb_* value is evaluated, the oxygen partial pressure in the capillary is determined using an inverted ODC. Both relationships are of the following form [10,11,12,37]:

(4) $$\begin{array}{ccc} S_{Hb}(P_{b})=\frac{P_{b}^{n}}{P_{b}^{n}+P_{50}^{n}} & \to & P_{b}=P_{50}{\left(\frac{S_{Hb}}{1-S_{Hb}}\right)}^{\frac{1}{n}} \end{array}$$

where *n* denotes the Hill coefficient (the slope of the dissociation curve), while *P*_50_ is the oxygen pressure corresponding to 50% hemoglobin saturation. The ODC parameters vary with temperature (Bohr effect), which was also included in the model assuming the linear dependence of *P*_50_ and *n* on temperature based on the data from [38].

As already mentioned, the bioheat transfer model and the oxygen distribution model are linked through parameters related to blood flow: the perfusion coefficient *w* and the blood velocity in the capillary *u_b_*. The relationship is of the following form [7,37]:

(5) $$\begin{array}{ccc} w=\frac{\pi R_{c}^{2}u_{b}}{\pi R_{t}^{2}L_{t}} & \to & u_{b}=wL_{t}\frac{R_{t}^{2}}{R_{c}^{2}} \end{array}$$

At the same time, for the perfusion coefficient, a polynomial function was assumed to describe its change depending on thermal damage to the tissue [5]:

(6) $$w(Arr)=\left\{\begin{array}{cc} \left(1+25Arr-260Arr^{2}\right)w_{0}, & 0\leq Arr\leq 0.1\\ \left(1-Arr\right)w_{0}, & 0.1<Arr\leq 1\\ 0, & Arr>1 \end{array}\right.$$

where *w*_0_ is the initial perfusion coefficient, while the coefficients in this function are defined in the way that maps the phenomena that occur in the tissue during the increase in temperature (the initial increase in perfusion during vasodilation) and the subsequent thermal damage (the decrease in perfusion resulting from rupture of the vasculature) [5].

*Arr* is the degree of thermal damage to the tissue expressed by the Arrhenius integral [4,17,25]:

(7) $$Arr(x,t^{F})=\int_{0}^{t^{F}}A\exp\left[-\frac{E}{RT(x,t)}\right]dt$$

where *R* [J mol^−1^ K^−1^] is the universal gas constant, *E* [J mol^−1^] is the activation energy, and *A* [s^−1^] is the preexponential factor. As a criterion for tissue necrosis, integral values of *Arr* = 1 and *Arr* = 4.6 are used, which correspond to a probability of 63% and 99% of cell death at a specific point **x**. It should also be noted that a change in perfusion coefficient will cause a change in blood velocity in the capillary according to Formula (5).

The bioheat transfer model takes into account the possibility of changing the thermophysical parameters with increasing temperature. The thermal conductivity λ is assumed to be temperature-dependent (temperature in Kelvin), while volumetric specific heat *c* is treated as dependent on thermal conductivity [4,13]:

(8) $$\lambda (T)=0.6489+0.0427\mathrm{atan}\left[0.0252(T-315.314)\right]$$

(9) $$c(\lambda )=\left(3.385\lambda +2.17\right)\cdot 10^{6}$$

### 2.2. Sensitivity Analysis of the Oxygen Distribution Model

As already mentioned, a certain problem in modeling phenomena occurring in living organisms is the variety of tissue parameter estimates. One method to account for this type of variability is sensitivity analysis. In this work, the direct method is used, in which the set of equations describing the process is differentiated with respect to the parameter to which the sensitivity analysis is performed. This means that for each parameter for which a sensitivity analysis is carried out, an additional task is solved. And, as a result, the so-called sensitivity functions are obtained [32,39,40,41]. In the case of the sensitivity analysis of the current oxygen distribution model, the appropriate transformation of all formulas that constitute this model is required, i.e., Formulas (2)–(4). This will be presented in the work and will be carried out for the seven parameters of the model under consideration. They are the following:

- Krogh coefficient *K_t_* and oxygen demand *M*_0_ (radial direction model, (2));
- Mass transfer coefficient *k* (radial and axial directions models, (2) and (3));
- Oxygen carrying a capacity of blood κ*_b_* and blood velocity in capillary *u_b_* (axial direction model, (3));
- The Hill coefficient *n* and the oxygen pressure corresponding to 50% hemoglobin saturation *P*_50_ (Hill oxygen dissociation curve, (4)).

Assuming that the sensitivity analysis is performed for *w* = *w*_0_, i.e., thermally intact tissue, and assuming that *p_s_* = *K_t_*, *M*_0_, *k*, κ_b_, *u_b_*, *n*, *P*_50_ (*s* = 1, …, 7), the sensitivity functions were assumed to be of the following form:

(10) $$\begin{array}{ccc} G_{s}=\frac{\partial S_{Hb}}{\partial p_{s}} & U_{bs}=\frac{\partial P_{b}}{\partial p_{s}} & U_{ts}=\frac{\partial P_{t}}{\partial p_{s}} \end{array}$$

To obtain these functions, the equations comprising the oxygen distribution model (2)–(4) were differentiated with respect to the parameter *p_s_*.

Differentiating Equation (2) by the parameter *p_s_*,

(11) $$\frac{\partial }{\partial p_{s}}\left[K_{t}\frac{1}{r}\frac{dP_{t}}{dr}+K_{t}\frac{d^{2}P_{t}}{dr^{2}}\right]=\frac{\partial }{\partial p_{s}}\left[M_{t}(P_{t})\right]$$

one obtains

(12) $$\frac{\partial K_{t}}{\partial p_{s}}\frac{d^{2}P_{t}}{dr^{2}}+K_{t}\frac{\partial }{\partial p_{s}}\left(\frac{d^{2}P_{t}}{dr^{2}}\right)+\frac{1}{r}\frac{\partial K_{t}}{\partial p_{s}}\frac{dP_{t}}{dr}+\frac{1}{r}K_{t}\frac{\partial }{\partial p_{s}}\left(\frac{dP_{t}}{dr}\right)=\frac{\partial M_{t}(P_{t})}{\partial p_{s}}$$

Then, by performing the necessary transformations and using substitution (cf. Equation (2)),

(13) $$\frac{d^{2}P_{t}}{dr^{2}}=\frac{M_{t}(P_{t})}{K_{t}}-\frac{1}{r}\frac{dP_{t}}{dr}$$

the resulting equation is of the form

(14) $$K_{t}\frac{d^{2}}{dr^{2}}\left(\frac{\partial P_{t}}{\partial p_{s}}\right)+K_{t}\frac{1}{r}\frac{d}{dr}\left(\frac{\partial P_{t}}{\partial p_{s}}\right)=\frac{\partial M_{t}(P_{t})}{\partial p_{s}}-\frac{\partial K_{t}}{\partial p_{s}}\frac{M_{t}(P_{t})}{K_{t}}$$

and in its final form, using the sensitivity function (10), it is

(15) $$\begin{array}{cc} K_{t}\frac{1}{r}\frac{d}{dr}\left[r\frac{dU_{ts}}{dr}\right]=\frac{\partial M_{t}}{\partial p_{s}}-\frac{\partial K_{t}}{\partial p_{s}}\frac{M_{t}}{K_{t}}, & \frac{\partial M_{t}}{\partial p_{s}}=\frac{\frac{\partial M_{0}}{\partial p_{s}}P_{crit}P_{t}+\frac{\partial M_{0}}{\partial p_{s}}P_{t}^{2}+M_{0}P_{crit}U_{ts}}{{\left(P_{crit}+P_{t}\right)}^{2}} \end{array}$$

In order to obtain the appropriate form of the boundary condition for *r* = *R_c_*, it should be written in the form of (**n** is a normal vector)

(16) $$j_{t}=-K_{t}\frac{dP_{t}}{dr}n=\frac{k}{2\pi R_{c}}\left(P_{t}-P_{b}\right)$$

and also differentiated by the parameter *p_s_*:

(17) $$-\frac{\partial K_{t}}{\partial p_{s}}\frac{dP_{t}}{dr}n-K_{t}\frac{\partial }{\partial p_{s}}\left(\frac{dP_{t}}{dr}\right)n=\frac{1}{2\pi R_{c}}\frac{\partial k}{\partial p_{s}}\left(P_{t}-P_{b}\right)+\frac{k}{2\pi R_{c}}\left(\frac{\partial P_{t}}{\partial p_{s}}-\frac{\partial P_{b}}{\partial p_{s}}\right)$$

Next, after ordering the derivatives and assuming that

(18) $$J_{ts}=-\frac{\partial K_{t}}{\partial p_{s}}\frac{1}{K_{t}}j_{t}$$

the final form of the condition is

(19) $$J_{ts}=\frac{1}{2\pi R_{c}}\left(P_{t}-P_{b}\right)\left[\frac{\partial k}{\partial p_{s}}-\frac{\partial K_{t}}{\partial p_{s}}\frac{1}{K_{t}}k\right]+\frac{k}{2\pi R_{c}}\left(U_{ts}-U_{bs}\right)$$

On the other hand, the boundary condition for *r* = *R_t_* is of the form

(20) $$\frac{dU_{ts}}{dr}=0$$

Differentiating by the parameter *p_s_* the equation for the axial direction (3),

(21) $$\pi R_{c}^{2}\frac{\partial }{\partial p_{s}}\left(u_{b}\kappa_{b}\frac{dS_{Hb}}{dz}\right)=\frac{\partial }{\partial p_{s}}\left(-kP_{b}+kP_{t}\right)$$

one obtains

(22) $$\pi R_{c}^{2}\left[\frac{\partial }{\partial p_{s}}\left(u_{b}\kappa_{b}\right)\frac{dS_{Hb}}{dz}+u_{b}\kappa_{b}\frac{\partial }{\partial p_{s}}\left(\frac{dS_{Hb}}{dz}\right)\right]=-\frac{\partial k}{\partial p_{s}}\left(P_{b}-P_{t}\right)-k\left(\frac{\partial P_{b}}{\partial p_{s}}-\frac{\partial P_{t}}{\partial p_{s}}\right)$$

or, with use of the sensitivity function (10),

(23) $$\pi R_{c}^{2}\left[\left(\frac{\partial u_{b}}{\partial p_{s}}\kappa_{b}+u_{b}\frac{\partial \kappa_{b}}{\partial p_{s}}\right)\frac{dS_{Hb}}{dz}+u_{b}\kappa_{b}\frac{dG_{s}}{dz}\right]=-\frac{\partial k}{\partial p_{s}}\left(P_{b}-P_{t}\right)-k\left(U_{bs}-U_{ts}\right)$$

In addition, it should be taken into account that (cf. Equation (3))

(24) $$\frac{dS_{Hb}}{dz}=-\frac{k}{\pi R_{c}^{2}u_{b}\kappa_{b}}(P_{b}-P_{t})$$

so, in the end,

(25) $$\frac{dG_{s}}{dz}=\frac{1}{\pi R_{c}^{2}u_{b}\kappa_{b}}\left\{\left[\frac{k}{u_{b}\kappa_{b}}\left(\frac{\partial u_{b}}{\partial p_{s}}\kappa_{b}+u_{b}\frac{\partial \kappa_{b}}{\partial p_{s}}\right)-\frac{\partial k}{\partial p_{s}}\right]\left(P_{b}-P_{t}\right)-k\left(U_{bs}-U_{ts}\right)\right\}$$

and, the initial condition for *z* = 0 is of the form

(26) $$U_{bs}=0$$

The last relationship needed to perform the sensitivity analysis in the capillary subdomain and determine the *U_bs_* function is the differentiated form of the inverted Hill curve (cf. Equation (4)):

(27) $$\frac{\partial P_{b}}{\partial p_{s}}=\frac{\partial P_{50}}{\partial p_{s}}{\left(\frac{S_{Hb}}{1-S_{Hb}}\right)}^{\frac{1}{n}}+P_{50}\frac{\partial }{\partial p_{s}}\left[{\left(\frac{S_{Hb}}{1-S_{Hb}}\right)}^{\frac{1}{n}}\right]$$

or in its final form:

(28) $$U_{bs}=\frac{\partial P_{50}}{\partial p_{s}}{\left(\frac{S_{Hb}}{1-S_{Hb}}\right)}^{\frac{1}{n}}+P_{50}\left[\frac{1}{n}{\left(\frac{S_{Hb}}{1-S_{Hb}}\right)}^{\frac{1}{n}-1}\frac{G_{s}}{{\left(1-S_{Hb}\right)}^{2}}-\frac{1}{n^{2}}{\left(\frac{S_{Hb}}{1-S_{Hb}}\right)}^{\frac{1}{n}}\frac{\partial n}{\partial p_{s}}\ln\left(\frac{S_{Hb}}{1-S_{Hb}}\right)\right]$$

For the sake of completeness of information, the final form of the differentiated Hill curve (4) is the following form:

(29) $$G_{s}=\frac{\partial S_{Hb}}{\partial p_{s}}=\frac{P_{b}^{n}P_{50}^{n}\frac{\partial n}{\partial p_{s}}\left(\ln P_{b}-\ln P_{50}\right)-nP_{b}^{n}P_{50}^{n-1}\frac{\partial P_{50}}{\partial p_{s}}+nP_{b}^{n-1}P_{50}^{n}U_{\mathrm{bs}}}{{\left(P_{b}^{n}+P_{50}^{n}\right)}^{2}}$$

In summary, to determine the sensitivity functions for the one parameter of the oxygen distribution model, one should use the following formulas:

- Radial direction model: (15), (19), and (18);
- Axial direction model: (25) and (26);
- Inverted oxygen dissociation curve: (28).

Based on the sensitivity functions, one can additionally determine the change in the oxygen partial pressure due to the simultaneous perturbation of all parameters [32]:

(30) $$\Delta P=\sqrt{\sum_{i=1}^{7}{\left(U_{s}\Delta p_{s}\right)}^{2}}$$

### 2.3. Methods of Solution

**Figure 3:**
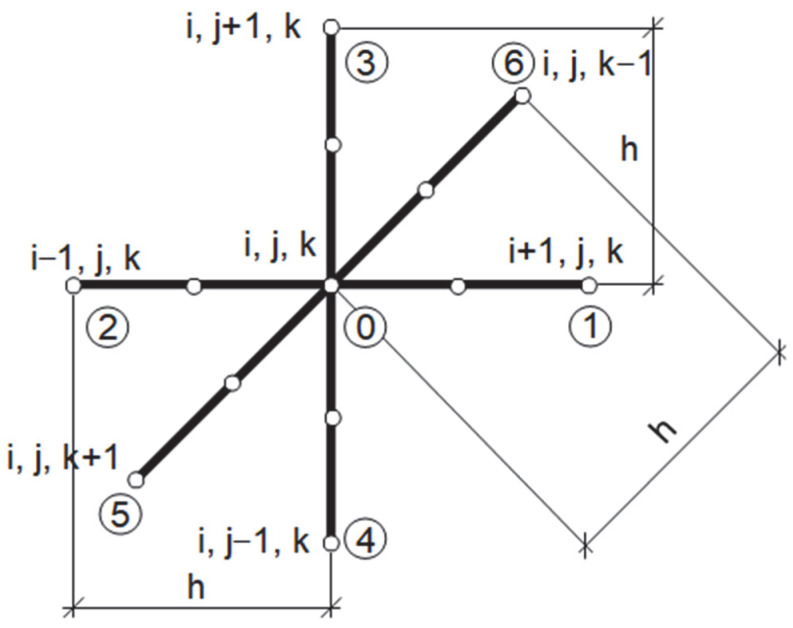
Seven-point stencil used in bioheat transfer problem.

The explicit scheme of the finite difference method that was applied in order to solve the bioheat transfer problem (cf. Equation (1)) uses the 7-point stencil shown in Figure 3, with the corresponding definitions of differential quotients (*h* denotes the grid step) [21]:

(31) $$\begin{array}{cc} {\left(\lambda \frac{\partial T}{\partial x}\right)}_{i+0.5,j,k}^{f-1}=\lambda_{01}\frac{T_{1}^{f-1}-T_{0}^{f-1}}{h} & {\left(\lambda \frac{\partial T}{\partial x}\right)}_{i-0.5,j,k}^{f-1}=\lambda_{02}\frac{T_{0}^{f-1}-T_{2}^{f-1}}{h}\\ {\left(\lambda \frac{\partial T}{\partial y}\right)}_{i,j+0.5,k}^{f-1}=\lambda_{03}\frac{T_{3}^{f-1}-T_{0}^{f-1}}{h} & {\left(\lambda \frac{\partial T}{\partial y}\right)}_{i,j-0.5,k}^{f-1}=\lambda_{04}\frac{T_{0}^{f-1}-T_{4}^{f-1}}{h}\\ {\left(\lambda \frac{\partial T}{\partial z}\right)}_{i,j,k+0.5}^{f-1}=\lambda_{05}\frac{T_{5}^{f-1}-T_{0}^{f-1}}{h} & {\left(\lambda \frac{\partial T}{\partial z}\right)}_{i,j,k-0.5}^{f-1}=\lambda_{06}\frac{T_{0}^{f-1}-T_{6}^{f-1}}{h} \end{array}$$

where

(32) $$\begin{array}{cc} \lambda_{0e}=\frac{2\lambda_{0}\lambda_{e}}{\lambda_{0}+\lambda_{e}}, & i=1,\ldots ,6 \end{array}$$

With the use of the differential quotients (31), for the central node of the stencil, we can write the following:

(33) $$\nabla \left(\lambda \nabla T\right)=\left[\frac{\partial }{\partial x}{\left(\lambda \frac{\partial T}{\partial x}\right)}_{0}^{f-1}+\frac{\partial }{\partial y}{\left(\lambda \frac{\partial T}{\partial y}\right)}_{0}^{f-1}+\frac{\partial }{\partial z}{\left(\lambda \frac{\partial T}{\partial z}\right)}_{0}^{f-1}\right]=\frac{1}{h^{2}}\sum_{e=1}^{6}\lambda_{0e}\left(T_{e}^{f-1}-T_{0}^{f-1}\right)$$

and the final equation for the central node of the stencil can be written in the following form:

(34) $$T_{0}^{f}=T_{0}^{f-1}+\frac{\Delta t}{ch^{2}}\sum_{e=1}^{6}\lambda_{0e}\left(T_{e}^{f-1}-T_{0}^{f-1}\right)+\frac{c_{B}w\Delta t}{c}(T_{B}-T_{0}^{f-1})+\frac{\Delta t}{c}Q_{met}$$

The solving equations for the boundary nodes are obtained in a similar manner.

The shooting method was used to solve the oxygen distribution model in the radial direction and related sensitivity analysis tasks [42,43,44]. The method involves replacing the boundary value problem (BVP) with the corresponding initial value problem (IVP). The boundary condition at the selected boundary Γ*_shoot_* of the domain under consideration is then used as the initial condition, while the second initial condition must be guessed. The obtained value of the solution “on the second boundary”, Γ*_target_*, of the domain must be compared with the given boundary condition there. The procedure is repeated until the value of the IVP solution agrees with the value of the boundary condition on Γ*_target_*. To solve IVP, one of the numerical methods for solving ODEs can be used, while approximate methods of solving equations are often used to determine subsequent guess values. In this work, a combination of the fourth-order Runge–Kutta method (solving IVPs) with the Newton method (determination of the guess values) was used, and *r* = *R_t_* was assumed as Γ*_shoot_*. This configuration of the shooting method was chosen due to the shorter calculation time, resulting mainly from the use of derivatives in the Newton method [42].

BVP can be written in a more general form as (cf. Equations (2), (15), (19), and (20))

(35) $$\begin{array}{cc} r\in \Omega_{t}: & K_{t}\frac{1}{r}\frac{d}{dr}\left(r\frac{dV_{t}}{dr}\right)=Q_{V}\\ r=R_{c}: & \frac{dV_{t}}{dr}+b_{1}V_{t}=b_{2}\\ r=R_{t}: & \frac{dV_{t}}{dr}=a \end{array}$$

where *V_t_* stands for *P_t_* or the corresponding sensitivity function *U_ts_*.

In the Newton method, consecutively guessed values α*_guess_* are determined as follows:

(36) $$\alpha_{guess}^{i+1}=\alpha_{guess}^{i}-\frac{\phi_{res}(\alpha_{guess}^{i})}{\phi_{res}^{\prime }(\alpha_{guess}^{i})}$$

where ϕ*_res_* and $\phi_{res}^{\prime }$ stand for differences checked on Γ*_target_* in the form

(37) $$\begin{array}{c} \phi_{res}(R_{c},\alpha_{guess})=V_{t}^{\prime }(R_{c},\alpha_{guess})+b_{1}V_{t}(R_{c},\alpha_{guess})-b_{2}\\ \phi_{res}^{\prime }(R_{c},\alpha_{guess})=Z^{\prime }(R_{c},\alpha_{guess})+b_{1}Z(R_{c},\alpha_{guess}) \end{array}$$

and

(38) $$\begin{array}{ccc} Z=\frac{\partial V_{t}}{\partial \alpha_{guess}} & Z^{\prime }=\frac{\partial V_{t}^{\prime }}{\partial \alpha_{guess}} & Q_{V}^{\prime }=\frac{\partial Q_{V}}{\partial \alpha_{guess}} \end{array}$$

Finally, two IVPs are solved simultaneously, the first resulting from the BVP in the form of (35), while the second determines the derivatives of *V_t_* relative to the guess value α*_guess_* in the following form:

(39) $$\begin{array}{cc} K_{t}V_{t}^{\prime \prime }+\;K_{t}\frac{1}{r}V_{t}^{\prime }=Q_{V} & K_{t}Z^{\prime \prime }+K_{t}\frac{1}{r}Z^{\prime }=Q_{V}^{\prime }\\ V_{t}(R_{t})=\alpha_{guess} & Z(R_{t})=1\\ V_{t}^{\prime }(R_{t})=a & Z^{\prime }(R_{t})=0 \end{array}$$

After the determination of the partial pressure in the tissue *P_t_* in the radial direction for a given node *m*, the saturation *S_Hb_* is calculated in the next node *m* + 1 based on the finite-difference-based formula (cf. Equation (3)) [13,21,25]:

(40) $$S_{Hb,m+1}=S_{Hb,m}-\frac{kh_{z}}{\pi R_{c}^{2}u_{b}\kappa_{b}}\left(P_{b,m}-P_{t,m}\right),\;m=0,1,\ldots ,nz$$

where *nz* is the number of nodes in the axial direction and *h_z_* is the step of the grid. Then, the partial pressure in the capillary *P_b_* in node *m* + 1 is also determined using Equation (4).

In a similar manner, the sensitivity function *G_s_* is calculated (cf. Equation (25)):

(41) $$G_{s,m+1}=G_{s,m}-\frac{h_{z}}{\pi R_{c}^{2}u_{b}\kappa_{b}}\left\{\left[\frac{k}{u_{b}\kappa_{b}}\left(\frac{\partial u_{b}}{\partial p_{s}}\kappa_{b}+u_{b}\frac{\partial \kappa_{b}}{\partial p_{s}}\right)-\frac{\partial k}{\partial p_{s}}\right]\left(P_{b,m}-P_{t,m}\right)-k\left(U_{bs,m}-U_{ts,m}\right)\right\}$$

## 3. Results and Discussion

### 3.1. Bioheat Transfer and Oxygen Distribution

A cube-shaped tissue domain with edge *L* = 1.5 cm was considered in the bioheat transfer problem, but due to symmetry, only a quarter of this area was taken into account with discretization of 76 × 76 × 151 nodes. Bioheat transfer analysis was performed with the following data: *c_B_* = 3.9962 [MJ m^−3^ K^−1^], *w*_0_ = 0.041 [s^−1^], *Q_met_* = 245 [W m^−3^], *T_B_* = 37 °C, *q*_0,*max*_ = 18,000 [W m^−2^], *t_exp_* = 22 [s], and *T_init_* = 37 °C, while for Arrhenius integral, *A* = 3.1 × 10^98^ [s^−1^], *E* = 6.27 × 10^5^ [J mol^−1^], and *R* = 8.314 [J mol^−1^ K^−1^] [7,25,41].

The following values of the parameters in the oxygen distribution model were assumed: *R_c_* = 2.5 [µm], *R_t_* = 25 [µm], *L_t_* = 500 [µm], *K_t_* = 4.1964 × 10^−14^ [(cm^2^ s^−1^) (mol cm^−3^ mmHg^−1^)], *M*_0_ = 2.9762 × 10^−7^ [mol cm^−3^ s^−1^], *P_crit_* = 1 [mmHg], *k* = 2.79 × 10^−13^ [(cm^2^ s^−1^) (mol cm^−3^ mmHg^−1^)], and κ*_b_* = 8.9286 × 10^−6^ [mol cm^−3^*_blood_*] [7,11,37]. For the change in the ODC, based on [38], for *T* = 37 °C, *P*_50_ = 27 and *n* = 2.57, while for *T* ≥ 44 °C, *P*_50_ = 35.9 and *n* = 2.45, and there was linear dependence between 37 and 44 °C.

The first step of analysis was associated with determining the temperature distribution, tissue damage, and damage-dependent perfusion coefficient, while in the second step, the values determined in this task for the selected node were used to solve the task related to oxygen distribution.

**Figure 4:**
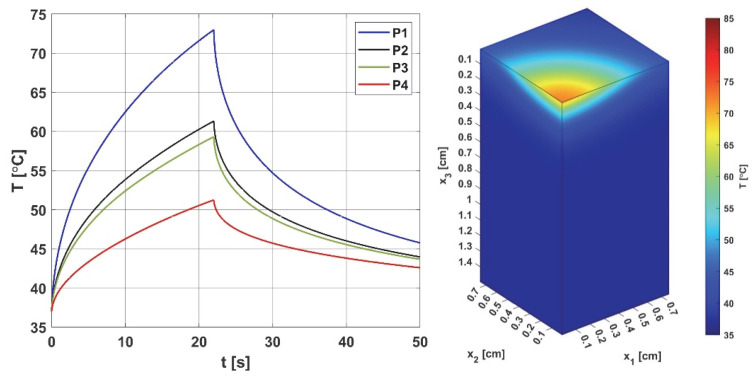
Histories of the temperatures at points P1–P4 (**left**) and the temperature distribution for *t* = 12.8 s (**right**). The history figure shows the increase in temperature at the considered points for the duration of the heat pulse (*t_exp_* = 22 s); for the final time of the performed simulation (50 s), the initial temperature of 37 ° C was not reached. The distribution is shown for time *t* = 12.8 s, for which maximum perfusion was reached at point P2 (cf. Figure 5 right).

**Figure 5:**
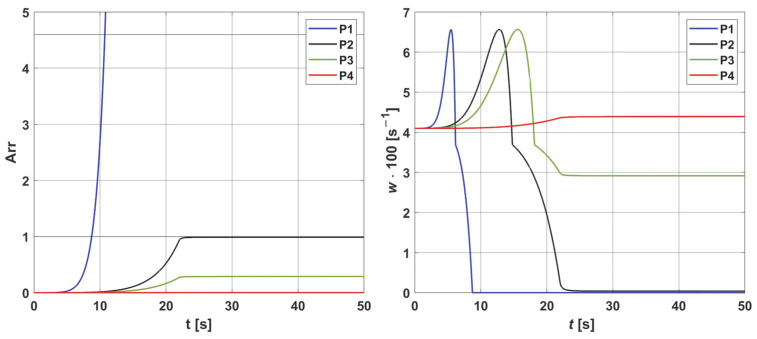
Histories of the Arrhenius integral (**left**) and the damage-dependent perfusion coefficient (**right**) at points P1−P4. For all points, an initial increase in perfusion resulting from vasodilation is evident. The necrosis criterion (*Arr* = 1) was reached only at points P1 and P2, meaning that the value of the perfusion coefficient decreased to zero at these points. For point P3, the achieved thermal damage value was *Arr* = 0.29, which means that perfusion was reduced to a value lower than the initial one (*w* = 0.029 [s^−1^]). At point P4, the damage reached a value less than *Arr* = 0.05 (the threshold for increased perfusion according to Equation (6), indicating elevated perfusion compared to the initial one (*w*_0_ = 0.041 [s^−1^])).

Figure 4 and Figure 5 are related to the bioheat transfer analysis. In Figure 4, the history of temperature at selected points of the domain (cf. Figure 2) and distribution of temperature in the domain under consideration are shown, while Figure 5 presents the courses of the Arrhenius integral and perfusion coefficient. The coordinates of the points are as follows [cm]: P1 (0, 0, 0.293), P2 (0, 0, 0.462), P3 (0, 0, 0.492), and P4 (0, 0, 0.641).

As can be seen (Figure 4), the temperature reaches values in the range of 51.24 °C (point P4) to 72.98 °C (point P1), and after the heat pulse stops (*t_exp_* = 22 s), it begins to decrease, but for the simulation time (50 s), the initial temperature of 37 °C was not reached. The left side of Figure 5, associated with the thermal damage model, calculated from the Arrhenius integral (Equation (7)), shows that thermal damage exceeded the necrosis threshold (*Arr* = 1) at points P1 and P2, which means that at these two points, the perfusion value dropped to zero after 9 and 22 s, respectively (Figure 5 right). At point P3, the *Arr* value reached 0.29, a phase of vascular damage in which the perfusion value was reduced from the initial value and was 0.029 [s^−1^]. Point P4 is the only one where the damage value reached a value below 0.05 (cf. Formula (6)), which means a slightly higher perfusion value compared to the initial value of *w*_0_ = 0.041 [s^−1^], with an increase in perfusion at points P1–P3, in the phases preceding the decrease in perfusion, is the maximum possible value. Based on the courses in Figure 5, point P2 was selected as the one where the Krogh cylinder under consideration (that is, the oxygen distribution model) was placed. At this point, the maximum perfusion value *w* = 0.066 [s^−1^] was reached for time *t* = 12.80 s.

To analyze oxygen distribution, it is necessary to know the velocity of the blood in the capillary *u_b_* (cf. Equation (5)) and the value of the partial pressure at the capillary inlet *P_b inlet_*. Two cases of this value are taken into account: *P_b inlet_* = 100 mmHg and *P_b inlet_* = 50 mmHg. The first corresponds to the conditions that prevail in normal and healthy tissue. In atypical and diseased tissues, especially cancerous ones, capillaries are often irregularly shaped, which is often associated with reduced *P_b inlet_* values, and therefore the second value of this parameter corresponds to just this kind of tissue [10].

**Figure 6:**
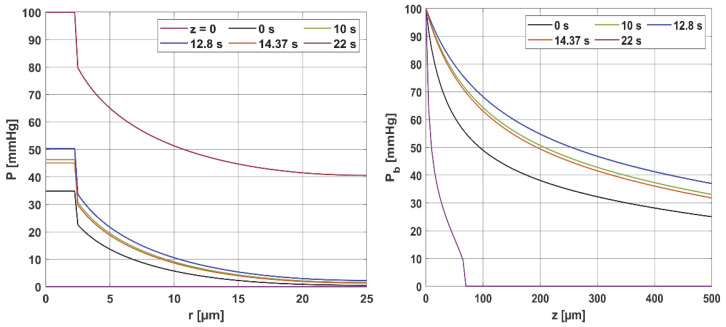
Distribution of the partial pressure in the radial and axial directions for selected time steps.

**Figure 7:**
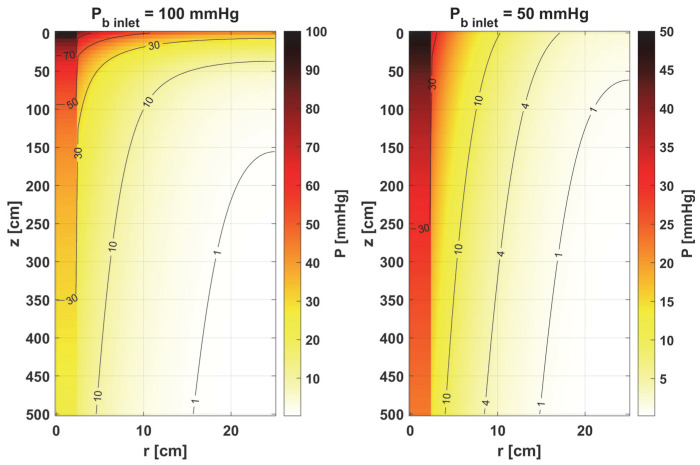
Comparison of the oxygen partial pressure in the Krogh cylinder for *P_b inlet_* = 100 mmHg and *P_b inlet_* = 50 mmHg.

Figure 6 presents the distribution of the partial oxygen pressure in the radial and axial directions for *P_b inlet_* = 100 mmHg (for the radial direction, the results correspond to *z* = 0 and *z* = *L_t_*/2), while in Figure 7, the comparison of the oxygen partial pressure in the entire Krogh cylinder for different values of the *P_b inlet_* is shown. Since *u_b_* (blood velocity in capillary) is calculated on the basis of the damage-dependent perfusion coefficient, this parameter has a quite visible effect on the level of oxygen partial pressure. Figure 6 shows that the curves for times 10, 12.8, and 14.37 s, for which the perfusion coefficient had values greater than the initial ones, also achieved higher oxygen partial pressure values. The highest curve corresponds to a time of 12.8 s, when the maximum value of the perfusion rate was recorded. Moreover, since the disappearance of perfusion occurred for a time of 22 s, for this time, it is visible that the hypoxia phenomenon occurs.

It should be mentioned that the calculations were carried out for temperature-dependent Hill ODC parameters. For comparison, calculations were also made for the case where the Bohr effect was not included. The maximum value of oxygen partial pressure for *z* = *L_t_* and a time of 12.8 s was then 31.67 mmHg, which is less than the results in Figure 6 (right); this value is 36.98 mmHg. Therefore, this is a discernible change. Also, for the case of fixed ODC parameters, for a time of 22 s, a decrease in *P_b_* to zero slightly further from the capillary inlet was observed (*z* = 90 µm, while in Figure 6 right for *z* = 70 µm). These results confirm the effect of temperature on the shape of the ODC.

Furthermore, the decrease in pressure at the capillary–tissue interface resulting from intravascular resistance is clearly visible (Figure 6 left). It should be noted that this phenomenon is not always taken into account in modeling the oxygen distribution. For many combinations of parameters, particularly those associated with typical tissues, this phenomenon does not produce such a large drop in pressure on the capillary wall. However, in the case we considered (Figure 7), the decrease in value between capillary and tissue pressures was similar in value to that of healthy tissue. A comparison of the pressure distribution shown in Figure 7 for the normothermia state shows that, obviously, with a reduced *P_b inlet_*, the pressure throughout the Krogh cylinder is reduced. The zone where *P_t_* < 1 mmHg has also increased. In many papers [13,37], this is considered as a hypoxic region.

### 3.2. Sensitivity Analysis

The point of using sensitivity analysis in modeling biological objects is, among other things, to take individual variability into account. On the other hand, sensitivity functions are able to illustrate where the greatest impact of changes in the parameter under consideration will occur, making it possible to identify at least the potential effects of such changes on the object under consideration. For example, when modeling oxygen distribution, there is no model to determine the degree of capillary damage, although, as already mentioned, it has been postulated that thermal damage models such as the Arrhenius scheme should include the effect of oxygen as some counteracting factor [24]. Furthermore, for most of the parameters related to the oxygen distribution model, there are no data regarding the dependence of these parameters on temperature and/or thermal damage. Basically, the exceptions to this are the parameters associated with the oxyhemoglobin dissociation curve (*n* and *P*_50_) (see Equation (4)), which depend on both temperature and pH, concentrations of CO_2_, and 2,3-DPG. Therefore, the purpose of carrying out a sensitivity analysis for the Krogh cylinder model in the current study will be to show the effect of changes in individual characteristics on the distribution of oxygen partial pressure in the domain under consideration. However, it should be noted that the sensitivity functions were determined for *w* = *w*_0_, which corresponds to the normothermic state (that is, tissue that is not thermally damaged).

**Figure 8:**
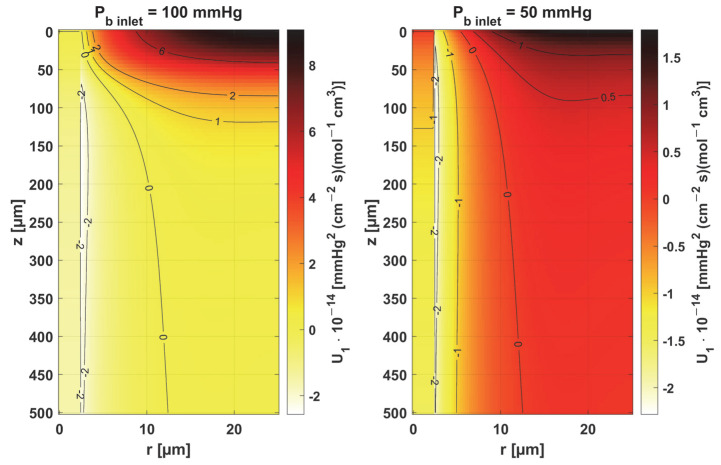
Sensitivity function of the Krogh coefficient *K_t_*.

**Figure 9:**
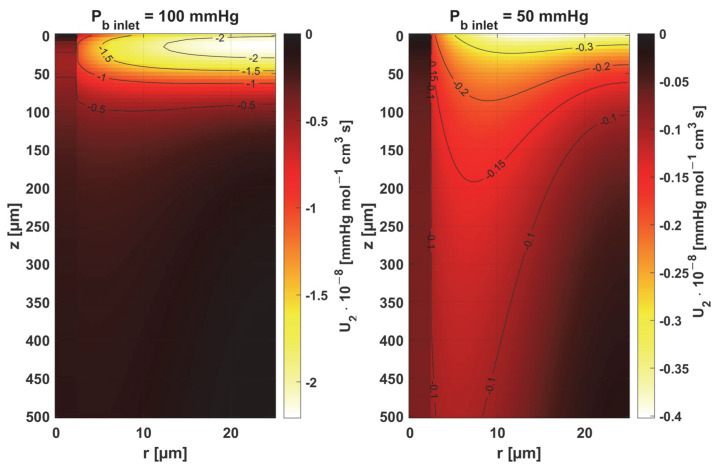
Sensitivity function of oxygen demand *M*_0_.

**Figure 10:**
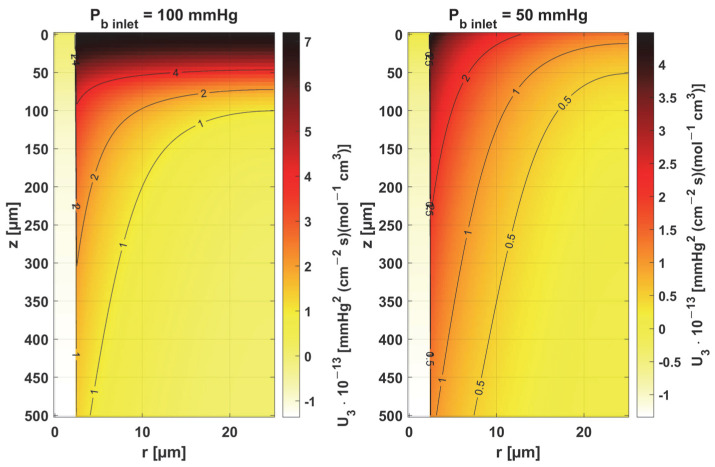
Sensitivity function of mass transfer coefficient *k*.

In Figure 8, Figure 9 and Figure 10, the sensitivity functions for the parameters related to the radial direction model are shown, namely the Krogh coefficient *K_t_*, the oxygen demand *M*_0_, and the mass transfer coefficient *k*. For all these parameters, a decrease in the value of the sensitivity function was observed for *P_b inlet_* = 50 mmHg; for *K_t_* and *M*_0_, the points with the maximum absolute value of this function moved closer to the capillary, for the latter parameter to the capillary–tissue interface. In the case of *K_t_*, for *r* = *R_c_*, there is a change in value due to the presence of this parameter also in the boundary condition (cf. Equation (2)). It may be mentioned that in publications on sensitivity analysis for bioheat transfer tasks, such shifts in sensitivity functions were often observed for thermal conductivity λ [45]. Also, for *K_t_*, for both *P_b inlet_* values, the level position for *U*_1_ = 0 is similar.

In the case of oxygen demand *M*_0_, it should be mentioned that for healthy tissues, under normothermic conditions, this value is equal to the supply of oxygen to the tissue. For diseased tissues, characterized by atypical capillary shapes, the value of *M*_0_ is often elevated due to oxygen deficiency in such tissues resulting from impaired oxygen supply. It should also be noted that the value of *M*_0_ adopted in the current work is quite high and corresponds to human skeletal muscle under conditions of good tissue oxygenation [37] compared to the values presented in the literature for various tissues [10,11].

For the mass transfer coefficient *k*, a clear difference can be seen in the level of the *U*_3_ function between the capillary and tissue, although the point of maximum absolute value of this function in both cases is *r* = *R_c_* and *z* = 0. This is due to the relationship of this parameter with intravascular resistance (cf. Figure 6), the value of which depends on the Sherwood number (*Sh*), a non-dimensional constant that depends on the oxygen transport process occurring within the vessel and reflects the particulate nature of blood. The Sherwood number for *R_c_* = 2.5 µm is in the range 1.5–3, and the mass transfer coefficient used in the work corresponds to *Sh* = 2.5 [37].

**Figure 11:**
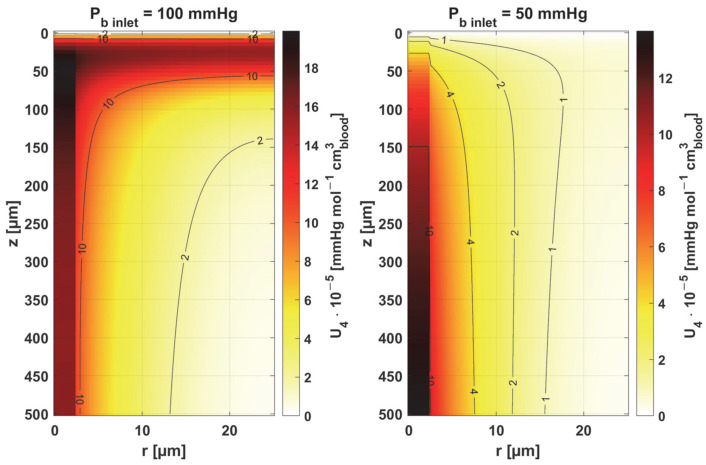
Sensitivity function of the oxygen-carrying capacity of blood, κ*_b_*.

**Figure 12:**
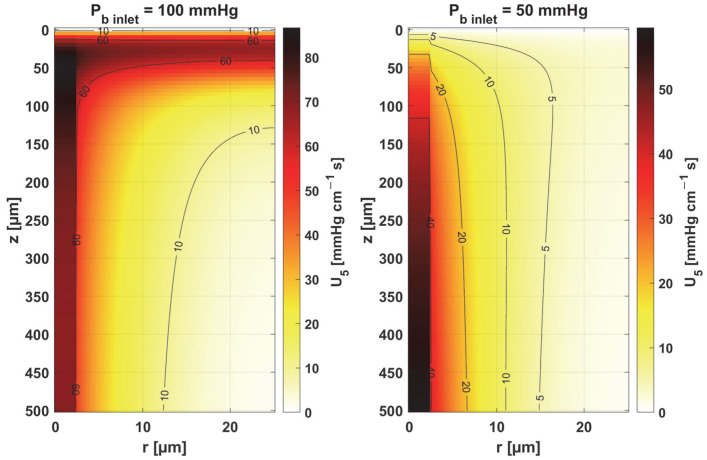
Sensitivity function of blood velocity in capillary, *u_b_*.

Figure 11 and Figure 12 show the sensitivity functions of the blood-related parameters, i.e., the oxygen-carrying capacity of blood κ*_b_* and the blood velocity in capillary *u_b_*. The resulting distributions are quite similar for both parameters, although different in values. For tissue with a normal *P_b inlet_*, the maximum values of the sensitivity functions are found in the anterior part of the capillary and tissue, while for a reduced *P_b inlet_*, there was a shift in the maximum values close to the outlet of the capillary. This change undoubtedly demonstrates the importance of both parameters in blood transport through the capillary, especially in atypical tissues. It should also be recalled that *u_b_* is a parameter linked to the bioheat transfer model, as its value is derived from the adopted value of the perfusion coefficient (cf. Equation (5)). In the literature, various values of capillary blood velocity are assumed for models based on the Krogh cylinder. For tumor tissues, very low values are sometimes given in the range of 0.02–0.03 cm s^−1^ [10,11], while the *u_b_* value calculated in the current work is close to 0.2 cm s^−1^.

**Figure 13:**
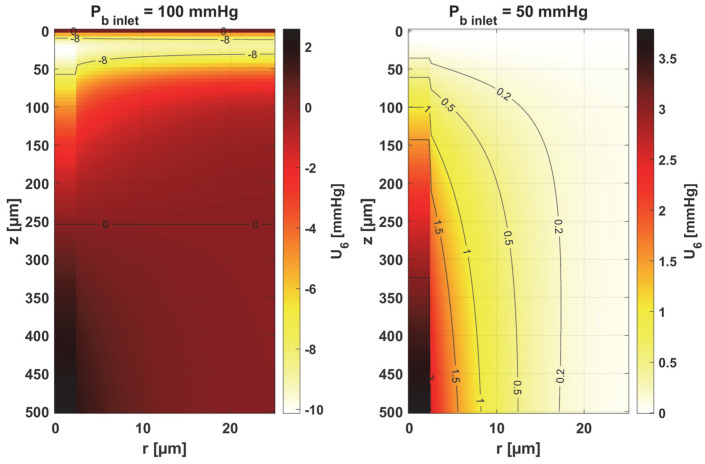
Sensitivity function of the Hill coefficient, *n*.

**Figure 14:**
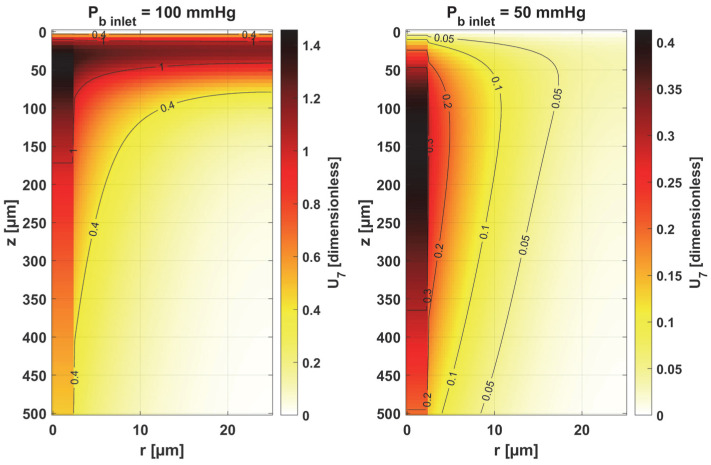
Sensitivity function of the oxygen pressure corresponding to 50% hemoglobin saturation, *P*_50_.

The sensitivity functions of the Hill ODC-related parameters are shown in Figure 13 and Figure 14. As already mentioned, these parameters show variability with temperature [11,12]. Although this issue is analyzed quite frequently in the literature [46,47], it should be noted that the available data generally relate to temperatures up to an upper limit of 44 °C [38]. From the point of view of the sensitivity function, the reduced pressure of *P_b inlet_* shifts the maximum absolute value of the sensitivity function to *z* = *L_t_* for the Hill coefficient and shifts the maximum near *z* = *L_t_*/2 for *P*_50_. It is easy to see that for *P_b inlet_* = 50 mmHg, the areas of values close to zero are quite large; also, the jump in value at *r* = *R_c_* is large. In the case of *P_b inlet_* = 100 mmHg, the change in *U*_6_ values from negative to positive occurs exactly in the middle of the capillary.

**Figure 15:**
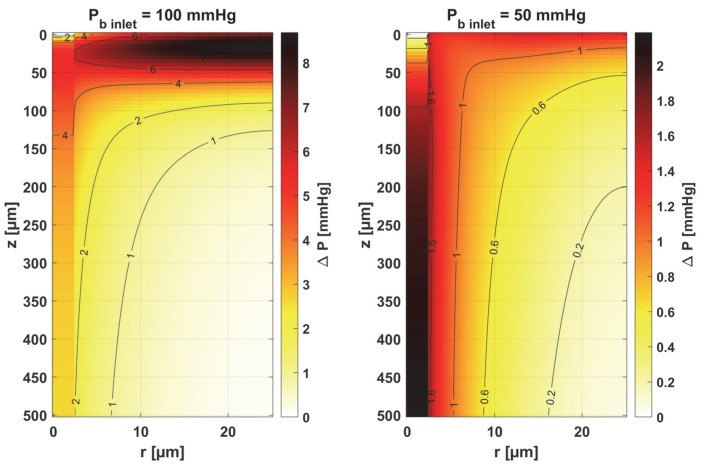
Distribution of change in oxygen partial pressure for simultaneous perturbation of all considered parameters (cf. Equation (30), Δ *p_s_* = *p_s_* × 10%).

Table 1 shows the values of the sensitivity functions multiplied by Δ *p_s_* = *p_s_* × 10% for the points where they are maximal. The results associated with the simultaneous perturbation of all considered parameters (cf. Equation (30)) are shown in Figure 15. It should be noted that the increments resulting from the sensitivity function of the individual parameters are not large, which is mainly due to the conditions for which the sensitivity analysis was performed, i.e., normothermia, the state before thermal damage, with fairly high values of blood velocity in the capillary. It is known that for such a condition, the partial oxygen pressure at the outlet of the capillary is around 30 mmHg (cf. Figure 6 and Figure 7) [37], even when the parameters are disturbed due to various reasons, to maintain normal vital processes.

Obviously, the increments, which were determined on a reduced *P_b inlet_* = 50 mmHg, are always lower. It should be noted that a living organism must ensure correct functioning, that is, in this particular case, the transport of oxygen to the tissue from the capillary, even for certain fluctuations in the parameter values (individual differences, but also disruptions in the functioning causing for disease or impact of temperature), so this type of result was rather expected. However, from the point of view of the shapes of the sensitivity function, it can be said that the areas where thermal damage has the first impact will undoubtedly be the capillary. Furthermore, the accumulation of the highest increment values at the capillary inlet for reduced *P_b inlet_* indicates that, for irregularly shaped capillaries, there may be interruptions in oxygen supply over a considerable length of the capillary. Since in therapies such as PDT (photodynamic therapy), oxygen is an essential factor in producing a cytotoxic effect, it can therefore be concluded that such interference will adversely affect reactions occurring during this kind of treatment [10,48].

As the results in Table 1 show, for a reduced *P_b inlet_* = 50 mmHg, the absolute values of the increments for the individual parameters are close to each other, although it should be noted that only for the mass transfer coefficient *k* is the maximum point situated in the same place as for *P_b inlet_* = 100 mmHg (*r* = *R_c_*, *z* = 0). In other cases, there was a shift of this point into the capillary, and for some parameters, there was a change in the sign of the increment (*K_t_*, *n*). For most parameters, the increments are in the interval of 1–2 mmHg, which, as already mentioned, is due to normothermic conditions. Only for *P_b inlet_* = 100 mmHg can larger increments be observed for the parameters associated with the radial direction model (*K_t_*, *M*_0_) and the parameters associated with the Hill ODC model.

An interesting case is the increments achieved for the blood velocity in the capillary, *u_b_*. Capillaries are smaller in diameter than RBCs (red blood cells), which are flattened to travel from the inlet to the outlet of the capillary, so it is known that *u_b_* is crucial from the point of view of blood flow through the capillary [12]. Meanwhile, the oxygen partial pressure increments derived from the sensitivity function *U*_5_ have average values compared to all the values included in Table 1.

The results for simultaneous perturbation of all parameters by 10% of their baseline value show that for *P_b inlet_* = 100 mmHg, it is possible to change the partial oxygen pressure to more than 8 mmHg in the tissue subdomain and to more than 5 mmHg in the capillary. These are, of course, higher values than those included in Table 1 for particular parameters. It should be noted that these values may be significant from the point of view of the phenomena that accompany tissue hypoxia, such as the accumulation of mitochondria near the capillary or the release of oxygen by myoglobin. The changes in the level of partial oxygen pressure caused by these phenomena are often much lower [11,12,37].

As is known, the Krogh coefficient is expressed as [10,11,12] follows:

(42) $$K_{t}=\alpha_{t}D_{t}$$

where α*_t_* [mol cm^−3^ mmHg^−1^] is the solubility of oxygen in the tissue, while *D_t_* [cm^2^ s^−1^] is the diffusion coefficient of oxygen in the tissue. We also determined the sensitivity functions for these two parameters. It turned out that their distributions in both cases have almost the same shape as the distribution of the *U*_1_ function (Figure 8), with differences in their values.

The value of the sensitivity function for α*_t_* is of the order of 10^10^, and for *D_t_* it is of the order of 10^5^, compared to the sensitivity function of *U*_1_, which is of the order of 10^14^. It should be noted, however, that for both *P_b inlet_* pressures considered, the maximum increments are almost identical for all three parameters and occur at the same points (cf. Table 1). From the point of view of the disruption of oxygen transport resulting from increased temperature, it should be mentioned that there are data that confirm the experimental variation in these parameters with temperature, showing that the decrease in oxygen solubility α*_t_* with increasing temperature is compensated for by an increase in the diffusion coefficient *D_t_* [49].

### 3.3. Results Verification

#### 3.3.1. Bioheat Transfer

To verify the correctness of the calculations for the bioheat flow problem (1), the following task was used:

(43) $$\begin{array}{cc} 0<x_{3}<L: & \begin{array}{c} \dot{T}(x_{3},t)=a\nabla^{2}T(x_{3},t)+b\left[T_{B}-T(x_{3},t)\right],\;a=\frac{\lambda }{c},\;b=\frac{c_{B}w}{c} \end{array}\\ x_{3}=0: & T(x_{3},t)=T_{0}\\ x_{3}=L: & T(x_{3},t)=T_{B}\\ t=0: & T(x_{3},t)=T_{B} \end{array}$$

for which there is an analytical solution of the form

(44) $$T(x_{3},t)=T_{B}+\frac{T_{0}-T_{B}}{2}\left[\exp\left(x_{3}\sqrt{\frac{b}{a}}\right)\mathrm{erfc}\left(\frac{x_{3}}{2\sqrt{at}}+\sqrt{bt}\right)+\left.\exp\left(-x_{3}\sqrt{\frac{b}{a}}\right)\mathrm{erfc}\left(\frac{x_{3}}{2\sqrt{at}}-\sqrt{bt}\right)\right]\right.$$

That is, in (1) *Q_met_* = 0 was assumed, and a transition was made from the heat flux condition to the Dirichlet condition.

**Figure 16:**
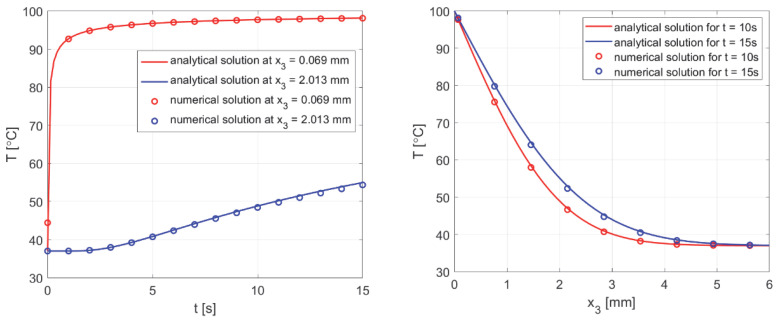
Comparison of the numerical solution of the bioheat transfer problem with the analytical solution (44).

The calculations were performed using data from [50], where a different analytical solution is presented for a similar task, but leading to exactly the same outcome. The results are shown in Figure 16, which managed to reproduce the results presented in [50] using (44) and the numerical solution of the current model.

#### 3.3.2. Oxygen Model

The correctness of the calculation for the oxygen distribution problem (2) was verified by the analytical solution that exists for the task with *P_crit_* = 0. It is in the following form:

(45) $$P_{t}(r)=P_{b}+\frac{M_{0}}{2K_{t}}\left[\frac{1}{2}\left(r^{2}-R_{c}^{2}\right)-R_{t}^{2}\ln\frac{r}{R_{c}}\right]+\frac{M_{0}\pi }{k}\left(R_{c}^{2}-R_{t}^{2}\right)$$

**Figure 17:**
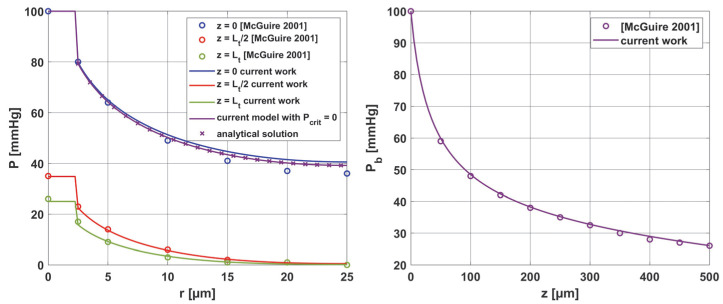
Comparison of results for the oxygen distribution model of the current work with analytical solution and results from [37].

In addition, the results were compared with the solutions obtained from the analysis presented in [37]. All comparisons were collected in Figure 17, with comparisons for the analytical solution (45) at *z* = 0. Good agreement was obtained for most curves; slight differences for comparison with [37] were observed only near the capillary inlet, for *r* = *R_t_*.

#### 3.3.3. Sensitivity Analysis

For the parameters *K_t_* and *M*_0_, it is possible to verify the correctness of the obtained sensitivity functions based on the analytical solution, which is obtained after differentiating (45) with respect to *p_s_*:

(46) $$U_{ts}(r)=U_{bs}+\frac{1}{2K_{t}^{2}}\left(K_{t}\frac{\partial M_{0}}{\partial p_{s}}-M_{0}\frac{\partial K_{t}}{\partial p_{s}}\right)\left[\frac{1}{2}\left(r^{2}-R_{c}^{2}\right)-R_{t}^{2}\ln\frac{r}{R_{c}}\right]+\frac{\pi }{k}\left(R_{c}^{2}-R_{t}^{2}\right)\frac{\partial M_{0}}{\partial p_{s}}$$

**Figure 18:**
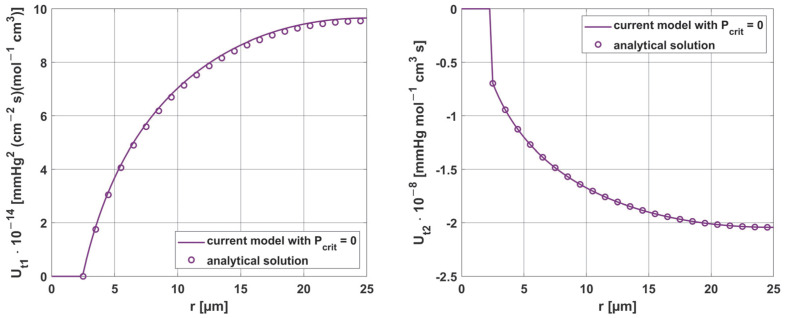
Comparison of the results of sensitivity analysis for the parameters *K_t_* and *M*_0_, using analytical solution (*z* = 0, *P_b inlet_* = 100 mmHg).

As can be seen in Figure 18, good agreement was achieved between the numerical solution obtained using the shooting method and the analytical solution.

Moreover, the results obtained for the sensitivity parameters *p_s_* were compared with the results considering parameter increments in the model using differential quotients. For all parameters, the relative error was within 0.01–0.02%, demonstrating the good accuracy of the methods used to solve these tasks. It should be noted here that, although the equations presented for the sensitivity analysis tasks in Section 2.2 are rather complicated in their general notation, for the individual parameters, some partial derivatives are equal to zero, which undoubtedly contributed to such good results obtained from the shooting method.

**Figure 19:**
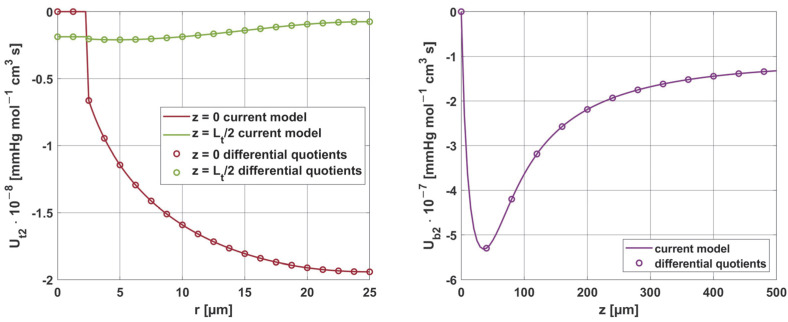
Comparison of the sensitivity functions of oxygen demand *M*_0_ obtained from the current model with calculations based on differential quotients.

**Figure 20:**
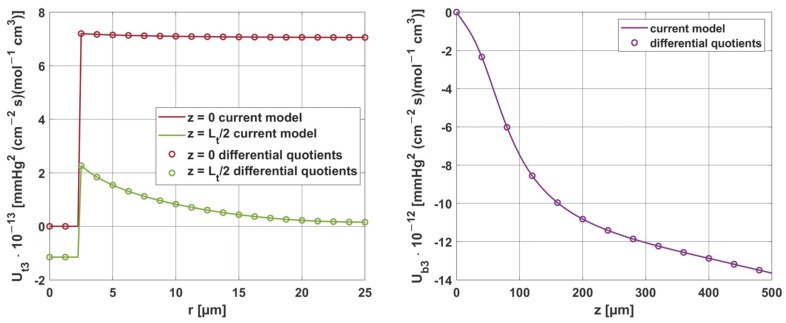
Comparison of the sensitivity functions of the mass transfer coefficient *k* obtained from the current model with calculations based on differential quotients.

Figure 19 and Figure 20 show the results for two selected parameters, oxygen demand *M*_0_ and mass transfer coefficient *k*, for the radial and axial directions.

## 4. Conclusions

The results of the calculations show the effect of elevated temperature on oxygen distribution in the tissue. An increase in thermal damage to the tissue affects the value of the perfusion coefficient (Figure 5) and therefore the blood velocity in the capillary, which is a parameter that combines models of bioheat transfer and oxygen distribution. A correspondingly low value of this parameter causes hypoxia. Note that the Bohr effect, i.e., the shift of the ODC to the right as a result of elevated temperature, was also included in the current work. It should be noted that the Bohr effect is fairly well described in the literature, but the availability of numerical data is still scarce, especially for ODC models other than the Hill model adopted here [12,13].

The key finding of this work is associated with the sensitivity analysis of the oxygen distribution model:

- Sensitivity functions were determined for the seven parameters present in the oxygen distribution model, for the normothermic state, as well as for two different values of the partial pressure at the capillary inlet corresponding to healthy and cancerous tissue (Figure 8, Figure 9, Figure 10, Figure 11, Figure 12, Figure 13 and Figure 14).
- The maximum increment values for each parameter were determined, assuming a 10% change in the parameter values (Table 1).
- The maximum increment value was determined for the simultaneous change of all considered parameters (Table 1, Figure 15).
- The most important features of the results are the following:
- The sensitivity functions for each parameter have completely different distributions for the case of the two *P_b inlet_* values considered.
- For tumor tissue (*P_b inlet_* = 50 mmHg), for each parameter and collectively, smaller increments were observed than for healthy tissue (*P_b inlet_* = 100 mmHg).
- For healthy tissue, the largest absolute values of increments were recorded for the parameters *K_t_*, *M*_0_, and *P*_50_, i.e., related to the radial direction model and ODC, while for cancer tissue, the largest increments values were quite close to each other and had different coordinates than for healthy tissue.
- In the case of blood-related parameters and ODC, for tumor tissue, the maximum increment points were located closer to the capillary outlet, indicating the importance of these parameters for hypoxic tissue.

Consideration of the above information can help to plan effective therapies using chemical and/or thermal effects on tissue [10,48]. There are also suggestions in the literature to consider oxygen as some counteracting factor to thermal tissue damage [24], which may also be relevant for a variety of therapies. Definitely, the use of sensitivity analysis methods can help in this type of analysis.

It is also worth noting that the model of bioheat transfer in the current work is the Pennes equation. Newer bioheat transfer equations like the dual-phase lag model can also be combined with oxygen distribution models [3,20,21], especially since the premise of the equation is to divide the tissue into so-called equivalent circles representing the area of tissue around the blood vessel. In light of the structure of the Krogh cylinder model, this offers interesting interpretative possibilities.

In conclusion, sensitivity analysis methods are still one of the best tools that exist to deepen our understanding of processes in living organisms that exhibit individual variation.

## Figures and Tables

**Table 1:** Increases in partial oxygen pressure expressed by multiplication of the sensitivity function by Δ *p_s_* = *p_s_* × 10% for points at which the increases are maximal. The values in the row ‘All parameters’ are obtained according to Equation (30).

	*P_b inlet_* = 100 mmHg			*P_b inlet_* = 50 mmHg		
Parameter	*U_s_* × Δ *p_s_* [mmHg]	*r* [µm]	*z* [µm]	*U_s_* × Δ *p_s_* [mmHg]	*r* [µm]	*z* [µm]
*K_t_*	3.8086	25	0	−0.9587	2.5	115
*M*_0_	−6.5718	25	15	−1.1972	14	0
*k*	2.0083	2.5	0	1.2516	2.5	0
*u_b_*	1.7771	1.25	50	1.2201	1.25	500
κ*_b_*	1.7771	1.25	50	1.2201	1.25	500
*n*	−2.6057	1.25	25	0.9736	1.25	500
*P*_50_	3.9371	1.25	40	1.1161	1.25	145
All parameters	8.6856	25	15	2.1871	1.25	500

## Data Availability

The original contributions presented in this study are included in the article. Further inquiries can be directed to the corresponding author.
